# Nec-1 Enhances Shikonin-Induced Apoptosis in Leukemia Cells by Inhibition of RIP-1 and ERK1/2

**DOI:** 10.3390/ijms13067212

**Published:** 2012-06-12

**Authors:** Weidong Han, Jiansheng Xie, Yong Fang, Zhanggui Wang, Hongming Pan

**Affiliations:** 1 Department of Medical Oncology, Sir Run Run Shaw Hospital, Zhejiang University School of Medicine, 3 East Qingchun Road, Hangzhou 310016, China; E-Mails: hanweidong1979@gmail.com (W.H.); fyzju@sina.com (Y.F.); wzg79@163.com (Z.W.); 2 The Cancer Institute, The Second Affiliated Hospital, Zhejiang University School of Medicine, 88 Jiefang Road, Hangzhou 310009, China; E-Mail: xjs85@126.com

**Keywords:** Necrostatin-1, receptor-interacting protein 1, shikonin, apoptosis, ERK1/2

## Abstract

Necrostatin-1 (Nec-1) inhibits necroptosis by allosterically inhibiting the kinase activity of receptor-interacting protein 1 (RIP1), which plays a critical role in necroptosis. RIP1 is a crucial adaptor kinase involved in the activation of NF-κB, production of reactive oxygen species (ROS) and the phosphorylation of mitogen activated protein kinases (MAPKs). NF-κB, ROS and MAPKs all play important roles in apoptotic signaling. Nec-1 was regarded as having no effect on apoptosis. Here, we report that Nec-1 increased the rate of nuclear condensation and caspases activation induced by a low concentration of shikonin (SHK) in HL60, K562 and primary leukemia cells. siRNA-mediated knockdown of RIP1 significantly enhanced shikonin-induced apoptosis in K562 and HL60 cells. Shikonin treatment alone could slightly inhibit the phosphorylation of ERK1/2 in leukemia cells, and the inhibitory effect on ERK1/2 was significantly augmented by Nec-1. We also found that Nec-1 could inhibit NF-κB p65 translocation to the nucleus at a later stage of SHK treatment. In conclusion, we found that Nec-1 can promote shikonin-induced apoptosis in leukemia cells. The mechanism by which Nec-1 sensitizes shikonin-induced apoptosis appears to be the inhibition of RIP1 kinase-dependent phosphorylation of ERK1/2. To our knowledge, this is the first study to document Nec-1 sensitizes cancer cells to apoptosis.

## 1. Introduction

Necroptosis is a recently discovered, regulated form of programmed necrosis initiated by the activation of tumor necrosis factor alpha (TNFα) and/or Fas that is distinct from caspase-dependent apoptotic cell death [1]. Necrostatin-1 (Nec-1) is a small molecule inhibitor originally identified in a chemical library screen as a potent and specific inhibitor of necroptosis. Usually, it does not protect against caspase-dependent apoptosis, or against other programmed cell death, such as autophagy [2]. Subsequent studies showed that Nec-1 allosterically inhibits the kinase activity of receptor-interacting protein 1 (RIP1) by interacting with the T-loop without affecting other functional domains, which is essential in death receptor (DR) triggered necroptosis [3], but has no effects on another pro-necrotic kinase receptor-interacting protein 3 (RIP3) [4]. Because of its relatively specific effect against the pro-necrotic activity of RIP1, Nec-1 has become a popular tool to investigate the role of necrosis in different experimental models of cell injury. Many reports indicate that Nec-1 provides *in vivo* protection in experimental models of ischemic brain injury [2], myocardial infarction [5], excitoxicity [6], and chemotherapy-induced cell death [7].

Shikonin (SHK) and its derivatives have been investigated as potential anti-cancer drugs for various aspects of cancer treatment over the last four decades [7–14]. We previously reported that shikonin and its analogues could induce necroptosis in breast cancer cells at all concentrations [7]. In HL60 and K562 cells, however, shikonin induced a dominant apoptosis at <2.5 μM, a dominant necroptosis at >10 μM. Interestingly, when HL60 and K562 cells were treated with shikonin (>10 μM) in the presence of Nec-1, we found that the necroptosis was switched to apoptosis [15]. These results indicated that apoptosis and necroptosis may function as reciprocal backup mechanisms of cellular demise.

The specificity of Nec-1 inhibiting necroptosis has been well established [2]. Nec-1 specifically inhibits the kinase activity of RIP1 and has no effect on the apoptotic signaling pathway. Previous studies have shown that RIP1 is crucial for activating NF-κB and production of reactive oxygen species (ROS) [16]. Moreover, under certain conditions, RIP1 is also involved in activating mitogen activated protein kinases (MAPKs), such as p38 MAPK, JNK and ERK [16]. It remains elusive which domain in RIP1 is essential for the activation of downstream signaling. It is also known that NF-κB, ROS and MAPKs play important roles in apoptosis signaling. Given that Nec-1 can inhibit phosphorylation of RIP1, we then asked whether Nec-1 affects the apoptotic signaling pathway. Shikonin was particularly chosen in our experiments due to its unique activity in death mode induction.

In the current study, we discovered that Nec-1 enhanced shikonin-induced apoptosis in the human leukemia cell lines K562 and HL60, as well as in primary leukemia cells. Further investigation indicated that Nec-1 enhanced shikonin-induced apoptosis through inhibition of RIP1 and ERK1/2 activation.

## 2. Results and Discussion

### 2.1. Results

#### 2.1.1. Nec-1 Enhances Shikonin-Induced Apoptosis in both Leukemia Cell Lines and Primary Leukemia Cells

**Figure 1:**
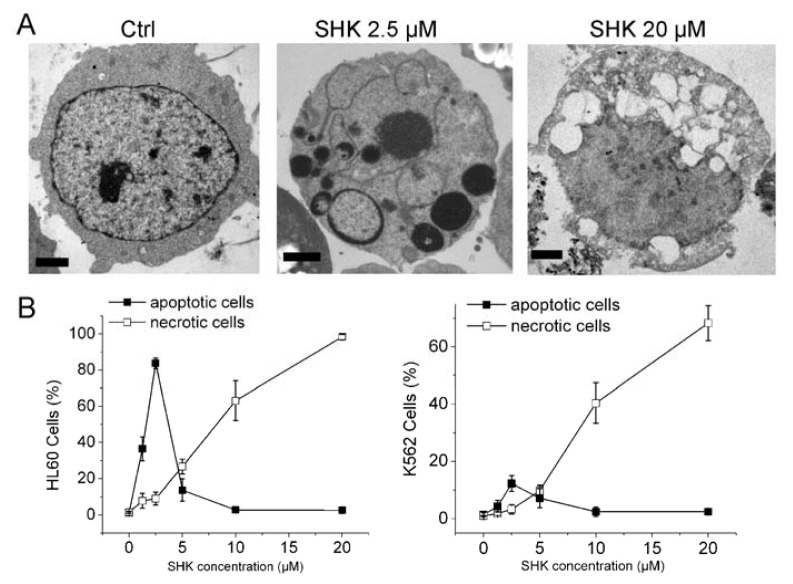

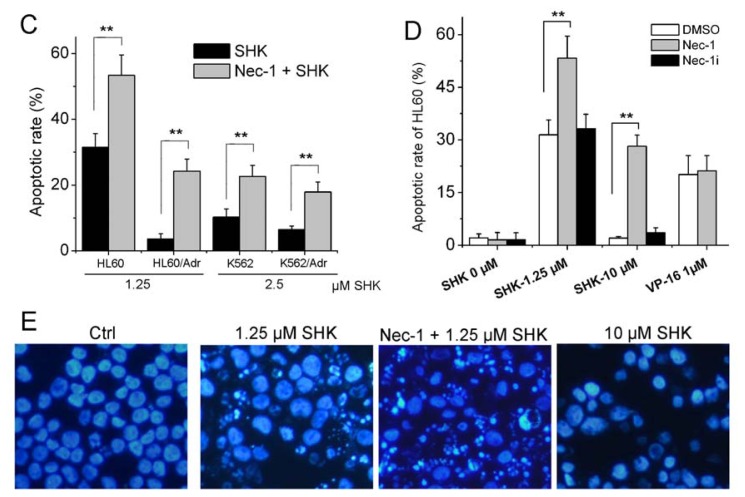
Nec-1 enhances shikonin-induced apoptosis in leukemia cells. (**A**) K562 cells were treated with various concentrations of shikonin for 12 h. Transmission electron micrograph showing that shikonin induced a typical apoptotic morphology at 2.5 μM, and a feature of necroptosis at 20 μM. Bar = 2 μm; (**B**) Cells were incubated with varying concentrations of shikonin for 12 h. Total cell death was measured by Vital dye exclusion assay and Hoechst-staining; (**C**) HL60, HL60/Adr, K562 and K562/Adr cells were treated with 1.25 or 2.5 μM shikonin for 12 h in the absence or presence of 60 μM Nec-1. Cells apoptotic rate was determined as described in Materials and Methods; (**D**) HL60 cells were treated with 1.25, 10 μM shikonin, or 1 μM VP-16 for 12 h in the absence or presence of 60 μM Nec-1 or Nec-1i. Cells apoptotic rate was determined as described in Materials and Methods. (**E**) Primary leukemia cells were treated with shikonin for 12 h in the presence or absence of Nec-1, and nuclei were stained by hoechst. Data are mean ± SD or representative of at least three independent experiments, and analyzed by Student’s *t* test. ** *p* < 0.01 compared with SHK treated group.

K562 and HL60 cells were incubated in the presence of shikonin for 12 h and subjected to morphological examinations. As previously reported, at low concentration of shikonin (1.25 or 2.5 μM), cells had morphology typical of apoptosis (chromatin margination and nuclear fragmentation). When the concentration of shikonin was raised up to 10 or 20 μM, cells exhibited no apoptotic nuclear characteristics, but severe vacuolation, massive mitochondria damage, many autophagosomes, indicating the occurrence of necrosis (Figure 1A,B and our published data in *Apoptosis* [15]).

We then treated HL60 and K562 cells with shikonin in the presence or absence of Nec-1 for 12 h, and counted the population of dead cells by Hoechst 33342 and trypan blue assays as previously described [15]. To our surprise, Nec-1 significantly enhanced low concentration-shikonin-induced apoptosis in leukemia cells. As shown in Figure 1C, when the cells were treated with SHK alone, the apoptotic rate of HL60 and K562 reached 31.4 ± 4.2 and 10.2 ± 2.5, respectively. In contrast, the apoptotic rate increased to 53.3 ± 6.2 and 22.7 ± 3.3, respectively, when the cells were treated with Nec-1 and SHK combined. Adriamycin-resistant HL60 cells (HL60/Adr) and K562 cells (K562/Adr) are characterized by overexpression of multidrug resistance-associated protein 1 (MRP1) or P-glycoprotein (P-gp). These cells are highly resistant to apoptosis induced by anticancer drugs [15]. We treated these two drug resistant cells with a low concentration of shikonin in the presence or absence of Nec-1. The results showed that the shikonin-induced apoptotic rate was much lower in resistant cells compared to its parent cells, and Nec-1 significantly enhanced shikonin-induced apoptosis in resistant cells (Figure 1C).

Consistent with our previous report, Nec-1 significantly converted 10 μM shikonin-induced necroptosis to apoptosis in HL60 cells. As an inactive control, Nec-1i had no effect on shikonin-induced apoptosis (Figure 1D).

The above results prompted us to investigate whether shikonin could induce primary human leukemia cell death in the same manner. Primary cells treated with a low concentration of shikonin (at 1.25 or 2.5 μM) exhibited apoptotic nuclear fragmentation. When treated with 10 μM shikonin, these primary cancer cells had morphology typical of necrosis without nuclear condensation (Figure 1E). Among primary cells from a total of 14 patients treated with 1.25 or 2.5 μM shikonin, 8 samples displayed a significantly increased apoptotic rate when cells were also treated with Nec-1 (Figure 1E and Table 1).

Collectively, these results demonstrated that the death mode of leukemias induced by shikonin was dose-dependent, *i.e.*, shikonin at <2.5 μM and >10 μM induced apoptosis and necroptosis, respectively. Nec-1 can convert high concentration shikonin-induced necroptosis to apoptosis, and interestingly, Nec-1 can enhance low-concentration-shikonin-induced apoptosis in leukemias.

#### 2.1.2. Nec-1 Enhances Shikonin-Induced Apoptosis through Increasing Caspases Activation in Leukemia Cells

**Figure 2:**
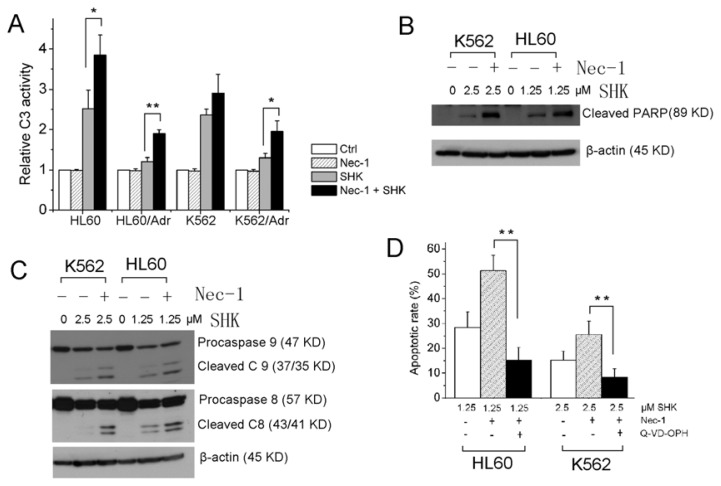
Nec-1 enhances shikonin-induced apoptosis through enhancing caspase activation in HL60 and K562 cells. (**A**) Cells were treated with shikonin in the presence or absence of Nec-1 for 12 h. Caspase-3 activation was measured using a Caspase-3/CPP32 Colorimetric Assay Kit; (**B**) HL60 and K562 cells were treated with shikonin in the absence or presence of Nec-1 for 12 h. The cleavage of PARP was detected by Western blot; (**C**) HL60 and K562 cells were treated with shikonin in the absence or presence of Nec-1 for 12 h. The activation of caspase 8 and 9 were reflected by the cleavage bands. β-actin served as an internal control; (**D**) HL60 and K562 cells were treated with 1.25 or 2.5 μM shikonin for 12 h in the absence or presence of 60 μM Nec-1 or 10 μM Q-VD-OPH. Cells apoptotic rate was determined as described in Materials and Methods. Data are mean ± SD or representative of at least three independent experiments, and analyzed by Student’s *t* test. * *p* < 0.05, ** *p* < 0.01 compared with SHK treated group.

Since leukemia cells treated with a low concentration of shikonin exhibited apoptotic nuclear fragmentation, we checked whether caspase-3, the major effector of caspases, and PARP, the main substrate of caspases, were involved. Activation of caspase-3 was assessed by using a caspase-3/CPP32 colorimetric assay kit. When treated with a low concentration of shikonin, HL60, HL60/Adr, K562 and K562/Adr cells exhibited caspase-3 activation. Moreover, the caspase-3 activity was remarkably augmented by Nec-1 (Figure 2A). Correspondingly, the cleavage of PARP (85 kDa fragment) was detected in cells treated with shikonin and was more pronounced by the Nec-1-SHK-combined treatment (Figure 2B), agreeable with the change in the amount of caspase-3. Then we checked the initiator caspases, caspase 8 and 9. Shikonin treatment led to activate both initiator caspases. The active cleavage bands of caspase 8 and 9 were increased in the presence of Nec-1 (Figure 2C). In order to validate if SHK-Nec-1-induced apoptosis is really dependent on caspase activity, HL60 cells were incubated with 10 μM Q-VD-OPH (a pan-caspase inhibitor) for 1 h followed by SHK treatment in the presence or absence of Nec-1. The results showed that Q-VD-OPH significantly inhibited SHK-Nec-1-induced apoptosis (Figure 2D).

**Figure 3:**
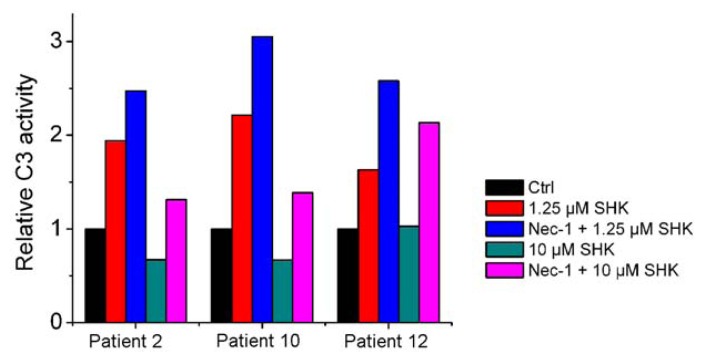
Nec-1 enhances shikonin-induced apoptosis via increasing caspase activation in primary leukemias. Primary leukemia cells were isolated and treated with shikonin in the absence or presence of Nec-1 for 12 h. Caspase-3 activation was determined as described in Materials and Methods. Three representative samples are shown out of 14 tested.

Similarly, Nec-1 enhanced shikonin-induced apoptosis could be duplicated using primary leukemia cells as reflected by enhanced caspase-3 activation (Figure 3).

#### 2.1.3. Knockdown of RIP1 Sensitizes Shikonin Induced Apoptosis

**Figure 4:**
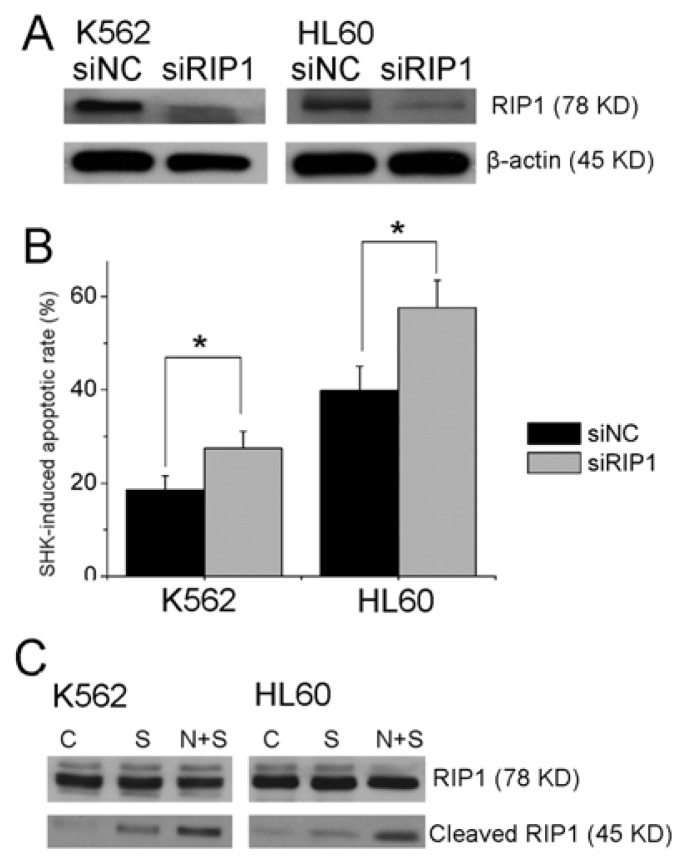
Knockdown of RIP1 sensitizes shikonin-induced apoptosis. (**A**) HL60 and K562 cells were transiently transfected with control siRNA or RIP1 siRNA. The knockdown efficiency was determined by Western blot 48 h post-transfection; (**B**) Cells transiently transfected with negative control or RIP1 siRNA were treated with 1.25 μM (HL60) or 2.5 μM (K562) SHK for 12 h. Cells apoptotic rate was measured as described in Materials and Methods; (**C**) HL60 and K562 cells were treated with shikonin in the absence or presence of Nec-1 for 12 h. The cleavage of RIP1 was detected by Western blot. Data are mean ± SD or representative of at least three independent experiments, and analyzed by Student’s *t* test. * *p* < 0.05.

As we know, Nec-1 is a specific inhibitor of necroptosis by targeting the RIP1 kinase domain [3]. Recent research indicated that Nec-1 might have RIP1 independent activity [17]. To rule out this possibility, we used genetic inhibition of RIP1 by transfection of siRNAs (Figure 4A). K562 cells transiently transfected with negative control siRNA or RIP1 siRNA were treated with 2.5 μM shikonin for 12 h. Apoptotic rates were determined by Hoechst staining. The results showed that knockdown of RIP1 increased the shikonin induced K562 apoptotic rate from 18.5 ± 3.0% to 27.5 ± 3.6%. The same sensitizing effect by knockdown of RIP1 was obtained in HL60 cells treated with 1.25 μM shikonin for 12 h (Figure 4B).

Because caspase-8 is responsible for RIP1 cleavage [18], we detected the cleavage of RIP1 by Western blot. Clearly, Nec-1 could enhance the cleavage of RIP1 induced by low concentration of shikonin in HL60 and K562 cells (Figure 4C).

#### 2.1.4. ERK1/2, but Not NF-κB and ROS Are Involved in the Nec-1 Enhancement of Apoptosis

**Figure 5:**
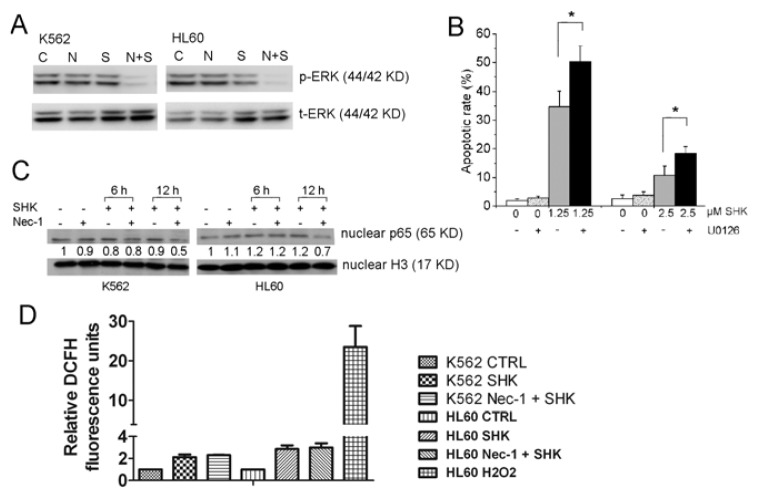
ERK1/2, but not NF-κB and ROS are involved in the Nec-1 enhancement of apoptosis. (**A**) HL60 and K562 cells were treated with shikonin in the absence or presence of Nec-1 for 12 h. The phosphorylation of ERK1/2 was detected by Western blot; (**B**) Cells were treated with SHK for 12 h in the presence or absence of 5 μM U0126. The apoptotic rate was measured as described in Materials and Methods; (**C**) HL60 and K562 cells were treated with shikonin in the absence or presence of Nec-1 for 0, 6 or 12 h. NF-κB activation was detected by Western blotting. Histone H3 served as an internal control of nuclear protein. Protein ratios were calculated following ImageJ densitometric analysis; (**D**) K562 and HL60 cells were treated with shikonin in the absence or presence of Nec-1 for 3 h and assayed for ROS using DCFH-DA. HL60 cells treated with 30% H_2_O_2_ were used as a positive control. Relative DCFH fluorescence units were calculated from fluorescence mean compared to the control group. Data are mean ± SD or representative of at least three independent experiments, and analyzed by Student’s *t* test. * *p* < 0.05 compared with SHK treated group.

To investigate the exact mechanism of RIP1 in sensitizing shikonin-induced apoptosis, we checked the phosphorylation status of ERK1/2. The results showed that shikonin treatment alone could slightly inhibit phosphorylation of ERK1/2 in K562 and HL60 cells. And the inhibitory effect on ERK1/2 was significantly augmented by Nec-1 (Figure 5A). Then we used U0126, which can inhibit activation of ERK1/2 by inhibition of MEK1/2, to check if inhibition of ERK1/2 could sensitize shikonin-induced apoptosis. As show in Figure 5B, the apoptotic rates in HL60 and K562 cells were 34.8 ± 5.4% and 10.7 ± 3.3%, respectively, after treatment with 1.25 or 2.5 μM shikonin. When combined with U0126, the apoptotic rates increased to 50.3 ± 3.1% and 18.3 ± 2.5% in HL60 and K562, respectively.

RIP1 plays a key role in nuclear factor-κB activation. We evaluated NF-κB activity by detecting translocated p65 in the nuclear fraction. Shikonin treatment alone failed to affect the nuclear p65. When cells were treated with shikonin in the presence of Nec-1, the nuclear p65 remained the same after 6 h of treatment, but significantly decreased after 12 h of treatment (Figure 5C). We further measured intracellular ROS level in K562 and HL60 cells treated with shikonin in the absence or presence of Nec-1. The results showed shikonin-treated cells had elevated intracellular reactive oxygen species, and Nec-1 had no effect on ROS accumulation in shikonin-treated cells (Figure 5D).

### 2.2. Discussion

Nec-1 is a specific inhibitor of necroptosis, and is considered to have no effect on apoptosis. In this study, we found that Nec-1 failed to enhance or attenuate apoptosis induced by etoposide (VP-16) in HL60 cells (Figure 1D). But our results showed that Nec-1 could enhance shikonin-induced apoptosis. To our knowledge, this is the first study to document that Nec-1 sensitizes cancer cells to apoptosis.

Shikonin and its derivatives have extensive anti-cancer activities as reported independently from a number of laboratories [8–11,13,14]. Our previous studies revealed that shikonin is a unique small molecule that could act on multiple cellular targets and trigger multiple death pathways, thus making it a promising candidate for cancer treatment, particularly in multi-drug resistance cancer cells [7,12,15]. In the present study, we found that shikonin had potent anti-cancer activities in primary leukemia cells. More importantly, both our previous data and current results suggest that shikonin could be used as a valuable tool to investigate the interaction between different death signaling pathways.

Nec-1 targets the RIP1 kinase step in the necroptosis pathway. RIP1 is a crucial molecule for signaling to NF-κB, MAPKs and ROS *etc*. [16,19]. It consists of an *N*-terminal kinase domain, an intermediate domain, a RIP homotypic interaction motif (RHIM), and a *C*-terminal death domain (DD) motif [16], and is a kinase with diverse and context-specific roles in inflammation, cell survival, and cell death [16,20]. RIP1 functions at the crossroads of the decision of cells to live or die upon exposure to several stress signals, such as cytokines, pathogen infections and genotoxic stress. Depending on the cellular context, this will lead to the activation of NF-κB, MAPKs, apoptosis or necrosis. Our results indicated that inhibition of RIP1 kinase activity by Nec-1 or knockdown of RIP1 expression using siRNA could promote shikonin-induced apoptosis in leukemia cells. The most pronounced change in the process of sensitizing shikonin-induced apoptosis by Nec-1 appears to be the inhibition of ERK1/2. ERK1/2 is a central component in the MAPK cascade. Generally, activation of ERK1/2 will promote survival in cancer cells [21]. For example, it can provide protection against chemotherapeutic cytotoxic drugs through regulating several members of the BCL-2 protein family to achieve cell survival [22,23]. In this study, we found that although shikonin treatment failed to increase the phosphorylation level of ERK1/2, Nec-1 can significantly inhibit the ERK1/2 in SHK-treated leukemia cells (Figure 5A). To test if RIP1-dependent ERK phosphorylation plays a direct role in Nec-1 sensitization to shikonin-induced apoptosis, we compared cell death in the absence or presence of U0126, a chemical inhibitor of the ERK-activating kinase. U0126 significantly augmented shikonin-induced apoptosis in HL60 and K562 cells (Figure 5B). Thus, the inhibition of ERK1/2 may be the key mechanism for promoting shikonin-induced-apoptosis by Nec-1. Consistent with our conclusion, recent studies indicate that the activation of ERK depends on the kinase activity of RIP1. Nec-1 could abolish TNFα + BV6-induced ERK phosphorylation [24].

NF-κB is widely used by eukaryotic cells as a regulator of genes that control cell proliferation and cell survival. When stimuli elicit the NF-κB pathway, the transcription factor subunits (p65 and p50) with exposed nuclear import signals translocate into the nucleus and bind regulatory elements that modulate gene transcription. So we set out to evaluate NF-κB activity by detecting translocated p65 in the nuclear fraction. Our data showed Nec-1 had no effect on p65 level in the nucleus of cells treated with shikonin for 6 h. When the treatment extended to 12 h, Nec-1 could significantly decrease the nuclear p65 (Figure 5C). It has been generally accepted that the kinase activity of RIP1 is dispensable for the activation of NF-κB [25,26], which seems to be inconsistent with the results that Nec-1 could inhibit p65 nuclear translocation in SHK treated leukemia cells. How to explain this inconsistency? Maybe inhibition of NF-κB by Nec-1 is indirect, because at the early stage of treatment (6 h), no difference of NF-κB p65 in the nucleus was found between SHK and SHK-Nec-1 treated cells (Figure 5C). When treated for a longer time (12 h), as described in Figure 2C, Nec-1 could enhance the activity of caspase-8, which counteracted NF-κB activation by cleaving RIP1 [27]. Therefore, augmenting the inhibition of NF-κB by Nec-1 could be caused by the enhancement of caspase-8 mediated cleavage of RIP1. Caspase-8-generated RIP1 fragments are incapable of inducing NF-κB activation [28]. Previous studies indicated that RIP1 controls downstream ROS production. We compared the intracellular ROS level in the absence and presence of Nec-1, and could not find a significant difference between SHK and SHK-Nec-1 treated cells (Figure 5D). The results indicated that ROS is not the key factor required for Nec-1 sensitization to shikonin-induced apoptosis.

Recent study showed that Nec-1 may exert RIP-1 independent effects. For example, Nec-1 can inhibit two other kinases, namely, PAK1 and PKAca [29]. So this RIP1 independent function of Nec-1 should be taken into consideration with care. Although our results indicated that knockdown of RIP1 by siRNA could also sensitize shikonin-induced apoptosis in leukemia cells, this evidence is inadequate. Other targets of Nec-1 should be further investigated.

## 3. Experimental Section

### 3.1. Reagents and Antibodies

Shikonin was purchased from J&K Chemical Ltd., China. Nec-1, Nec-1i, U0126, Q-VD-OPH were purchased from Sigma-Aldrich. Primary antibodies against cleaved PARP, non-phospho- or phospho-ERK1/2, RIP1, caspase 8 and 9, NF-κB p65, Histone H3 and β-actin were from Cell Signaling Technology, Inc. The secondary antibodies were HRP conjugated anti-rabbit and anti-mouse IgG (Cell Signaling Technology, Inc.).

### 3.2. Cell Cultures

HL60 and K562 cells were purchased from Cell Bank (Chinese Academy of Sciences). Cells were maintained in RPMI-1640 supplemented with 10% (*v*/*v*) FBS. Human leukemia multiple drug resistant (MDR) cell line HL60/Adr and K562/Adr were grown in RPMI-1640 containing 10% FBS and 0.5 μg/mL doxorubicin. Stock solutions of Nec-1, Nec-1i, U0126 and SHK were prepared in dimethyl-sulfoxide (DMSO, Sigma) and subsequently diluted with medium prior to use. The final concentration of DMSO was less than 0.1%.

### 3.3. Primary Human Leukemia Cells

Primary leukemia cells were collected upon informed consent and the approval of the hospital’s Institutional Review Board. Leukemia bone marrow specimens obtained were from the Department of Hematology, the First Affiliated Hospital, Zhejiang University School of Medicine. Enriched leukemia cell populations containing >95% tumor cells were prepared using a density gradient separation of samples (Ficoll-Hypaque, Sigma). The mononuclear cells were cultured in RPMI-1640 supplemented with 10% FCS. Viability on samples was >90% as determined by a trypan blue exclusion assay.

### 3.4. Treatment of Cells with Shikonin in the Presence or Absence of Nec-1

Unless otherwise stated, throughout this study, HL60 or HL60/Adr was treated with shikonin at 1.25 μM for 12 h; K562 or K562/Adr was treated with shikonin at 2.5 μM for 12 h. Nec-1 or Nec-1i was used at 60 μM for 1 h before shikonin treatment in all cellular assays.

### 3.5. Electron Microscopy

Treated cells were washed and fixed for 30 min with 2.5% glutaraldehyde. The samples were then treated with 1.5% osmium tetroxide, dehydrated with acetone and embedded in Durcupan resin. Thin sections were poststained with lead citrate and examined using the TECNAI 10 electron microscope (Philips, Holland) at 60 kV.

### 3.6. Vital Dye Exclusion Assay and Hoechst-Staining

Plasma membrane integrity of cells was tested by staining with 0.2% trypan blue (Sigma). The trypan blue positive cells were detected using a light microscope. For identification of cells with nuclear changes typical of apoptosis, cells were fixed, washed twice with PBS and stained with 5 μg/mL Hoechst 33,258 staining solution (Sigma-Aldrich) for 5 min at room temperature and observed by fluorescence microscopy using a 4′,6-diamidino-2-phenylindole (DAPI) filter. The apoptotic rate was determined by counting the cells with fragmented or condensed nuclei under a microscope at ×200 magnification on 5 random fields (counting ~50 cells/field) in each slide.

### 3.7. Measurement of Caspase-3 Activity

Caspase-3 activity was measured using a Caspase-3/CPP32 Colorimetric Assay kit (BioVision Research Products) according to the manufacturer’s instructions. Briefly, cell lysate from 10^6^ cells was incubated at 37 °C for 2 h with 200 μM DEVD-ρNA substrate. Samples were read at 400 nm with an ELX800 Micro Plate Reader (Bio-Tek Instruments, Inc.) and expressed as fold increase above basal level (DMSO-treated cells).

### 3.8. Western Blot Analysis

Western blotting was carried out as previously described [7]. The protein was applied to an appropriate concentration of SDS-polyacrylamide gel, transferred to a PVDF membrane, and then detected by the corresponding primary and secondary antibodies before visualization with a chemiluminescence kit (Pierce). Visualization was done with Image Quant LAS-4000 (Fujifilm, Tokyo, Japan) using image Multi-Gauge Software (Fujifilm, Tokyo, Japan). Band intensities were quantified using ImageJ software.

### 3.9. Small Interfering RNA Knockdown of RIP1

The RNAi duplex oligo-ribonucleotides were purchased from Invitrogen. The RNA sequences were as follows: (siRIP1) sense strand, 5′-GGA GCA AAC UGA AUA AUG AAG AGC A-3′; control siRNA oligonucleotide: 5′-CAG AGA GGA GGA AAG GAG ACG CAG G-3′. siRNAs were introduced into HL60 and K562 cells using a Nucleofection system (Amaxa) according to the manufacturer’s instructions. Cells were harvested for the following experiments 48 h after electroporation.

### 3.10. Reactive Oxygen Species Detection

Cells were plated in six-well plates and treated with shikonin in the presence or absence of Nec-1 at the designated intervals. The treated cells were incubated with phenol red-free RPMI-1640 (Invitrogen) containing 1 μM 6-carboxy-2′,7′-dichlorodihydrofluoresceindiacetate (Molecular Probes) in the dark for 30 min at 37 °C. Then, the cells were harvested and analyzed using a FACS Calibur (Becton Dickinson).

### 3.11. Statistical Analyses

Unless otherwise stated, data are expressed as the mean ± SD, and were analyzed by Student’s *t*-test.

## 4. Conclusions

In this study, we found that Nec-1 could promote low concentration-shikonin-induced caspase-dependent apoptosis in leukemia cell lines and primary leukemia cells. We discovered the mechanism of this sensitizing was due to the inhibition of RIP1 and ERK1/2 phosphorylation by Nec-1.

## Figures and Tables

**Table 1:** Nec-1 enhances shikonin-induced apoptosis in primary leukemia cells.

Patient No.	Sex	Age	Diagnosis	SHKconc. (μM)	Apoptotic rate (SHK)	Apoptotic rate (SHK + Nec-1)	*p* value (SHK *vs*. SHK + Nec-1)
1	M	74	AML	2.5	15.9 ± 2.5	24.3 ± 4.8	0.05669
2	F	49	AML	1.25	6.7 ± 3.7	20.6 ± 1.7	0.00398 **
3	M	24	AML	2.5	10.1 ± 4.5	18.8 ± 3.0	0.05162
4	M	28	CML	2.5	9.3 ± 2.8	15.2 ± 3.7	0.09042
5	F	54	CML	1.25	7.5 ± 3.2	15.4 ± 2.1	0.02372 *
6	M	33	CML	1.25	10.5 ± 2.4	19.9 ± 3.2	0.01594 *
7	F	35	CML	2.5	11.5 ± 1.9	18.5 ± 3.2	0.03051 *
8	M	43	CML	2.5	13.1 ± 4.6	18.6 ± 3.7	0.18326
9	M	53	AML	1.25	10.1 ± 1.0	13.3 ± 2.8	0.13721
10	F	18	AML	1.25	11.0 ± 3.6	24.8 ± 2.8	0.0065 **
11	M	55	CML	2.5	15.1 ±3.0	26.2 ± 4.0	0.01778 *
12	M	66	CML	2.5	13.8 ± 1.8	23.1 ± 2.8	0.00849 **
13	M	6	AML	1.25	23.3 ± 5.7	36.8 ± 7.2	0.0629
14	F	62	CML	1.25	35.8 ± 6.6	78.1 ± 7.7	0.00192 **

Note: Cells were incubated with 1.25 or 2.5 μM SHK for 12 h in the presence or absence of 60 μM Nec-1. Cells were harvested and assayed for Hoechst staining. Data represent means ± SD from triplicate of the same experiment. *t*-test

* *p* < 0.05;

** *p* < 0.01.
